# *COL4A5* and *LAMA5* variants co-inherited in familial hematuria: digenic inheritance or genetic modifier effect?

**DOI:** 10.1186/s12882-018-0906-5

**Published:** 2018-05-16

**Authors:** Konstantinos Voskarides, Gregory Papagregoriou, Despina Hadjipanagi, Ioanelli Petrou, Isavella Savva, Avraam Elia, Yiannis Athanasiou, Androulla Pastelli, Maria Kkolou, Michalis Hadjigavriel, Christoforos Stavrou, Alkis Pierides, Constantinos Deltas

**Affiliations:** 1 Molecular Medicine Research Center, Department of Biological Sciences, University of Cyprus, 1, University Avenue, 2109 Nicosia, Cyprus; 2 Medical School, University of Cyprus, Nicosia, Cyprus; 3 Department of Pediatric Nephrology, Archbishop Makarios III Hospital, Nicosia, Cyprus; 4 Department of Nephrology, Nicosia General Hospital, Nicosia, Cyprus; 5 Department of Nephrology, Larnaca General Hospital, Larnaca, Cyprus; 6 Department of Nephrology, Evangelismos Hospital, Pafos, Cyprus; 7 Hippocrateon Hospital, Nicosia, Cyprus; 8 College of Medicine, Qatar University, Doha, Qatar

**Keywords:** Digenic inheritance, Modifier gene, Familial hematuria, Renal cysts, Collagen IV, FSGS, Thin Basement Membrane Nephropathy (TBMN), Kidney disease, Laminin alpha 5, Alport syndrome, Synaptopodin, Metalloproteinase

## Abstract

**Background:**

About 40–50% of patients with familial microscopic hematuria (FMH) caused by thin basement membrane nephropathy (TBMN) inherit heterozygous mutations in collagen IV genes (*COL4A3*, *COL4A4*). On long follow-up, the full phenotypic spectrum of these patients varies a lot, ranging from isolated MH or MH plus low-grade proteinuria to chronic renal failure of variable degree, including end-stage renal disease (ESRD).

**Methods:**

Here, we performed Whole Exome Sequencing (WES) in patients of six families, presenting with autosomal dominant FMH, with or without progression to proteinuria and loss of renal function, all previously found negative for severe collagen IV mutations. Hierarchical filtering of the WES data was performed, followed by mutation prediction analysis, Sanger sequencing and genetic segregation analysis.

**Results:**

In one family with four patients, we found evidence for the contribution of two co-inherited variants in two crucial genes expressed in the glomerular basement membrane (GBM); *LAMA5*-p.Pro1243Leu and *COL4A5-*p.Asp654Tyr. Mutations in *COL4A5* cause classical X-linked Alport Syndrome, while rare mutations in the *LAMA5* have been reported in patients with focal segmental glomerulosclerosis. The phenotypic spectrum of the patients includes hematuria, proteinuria, focal segmental glomerulosclerosis, loss of kidney function and renal cortical cysts.

**Conclusions:**

A modifier role of *LAMA5* on the background of a hypomorphic Alport syndrome causing mutation is a possible explanation of our findings. Digenic inheritance is another scenario, following the concept that mutations at both loci more accurately explain the spectrum of symptoms, but further investigation is needed under this concept. This is the third report linking a *LAMA5* variant with human renal disease and expanding the spectrum of genes involved in glomerular pathologies accompanied by familial hematurias. The cystic phenotype overlaps with that of a mouse model, which carried a *Lama5* hypomorphic mutation that caused severely reduced Lama5 protein levels and produced kidney cysts.

**Electronic supplementary material:**

The online version of this article (10.1186/s12882-018-0906-5) contains supplementary material, which is available to authorized users.

## Background

Hereditary hematuric diseases comprise a genetically and clinically heterogeneous group of conditions, a common feature of which is microscopic hematuria (MH) since early childhood. The most frequent pathological entity is thin basement membrane nephropathy (TBMN) [1, 2], with an estimated population prevalence of 0.3–1% [3, 4]. About 40–50% of familial TBMN are explained by heterozygous mutations in the *COL4A3* and *COL4A4* genes, which encode for the α3 and α4 chains of collagen IV, the most abundant component of GBM [5]. A much more severe and progressive glomerulopathy, which also presents with MH since childhood is Alport syndrome, caused by either mutations in the *COL4A3/A4* genes or mutations in the X-linked *COL4A5* gene [2, 6]. Mutations in *CFHR5*, a gene playing a role in the regulation of the alternative pathway of complement, in *FN1* (fibronectin 1), or in *MYH9* (heavy chain of myosin 9), comprise rarer genetic causes of hereditary hematurias [1, 6–8].

Although TBMN, caused by *COL4A* mutations, was considered for many years to be a benign condition accompanied by excellent prognosis on long follow-up, several publications, including ours as early as 2007, convincingly showed that this is not the case [9–11]. This is based on results showing that a variable subset of patients will develop proteinuria and focal segmental glomerulosclerosis (FSGS) and progress to chronic renal failure, even end-stage renal disease (ESRD) [12]. In a Cypriot cohort, up to 30% of TBMN patients with known mutations reached ESRD, at an average age of 56-years. To date, these results have been verified by multiple groups [13–17]. The exact mechanisms for this adverse outcome remain unknown but the role of genetic modifiers has been implicated [18–22]. A significant feature of heterozygous *COL4A* mutations is the broad phenotypic heterogeneity, while the clinical outcome is at times better described as later-onset Alport-related nephropathy (LOAN) [23]. Some authors also use the diagnosis of autosomal dominant Alport syndrome.

Based on available data there must be more genes that confer FMH when mutated [11, 24]. Whole-exome sequencing (WES) is one of the best modern genetic approaches for gene discovery. Here we report the analysis of 6 families by (WES) and the finding of two variants co-inherited in two genes, the *COL4A5* and the *LAMA5* genes. Invoking this digenic inheritance can better explain the spectrum of symptoms observed in some patients, than the variant at each one locus alone [25].

## Methods

**Fig. 1:**
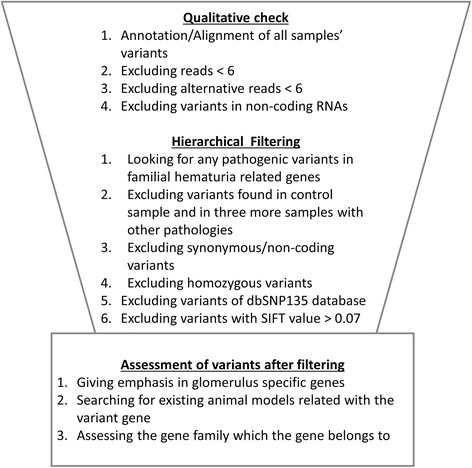
Hierarchical filtering that we followed for mining the Whole Exome Sequencing data

Six families segregating FMH in at least two generations (autosomal dominant inheritance) were thoroughly studied. Some patients also had proteinuria, renal impairment or ESRD. Renal biopsy showed FSGS and TBMN in two of six families (Families CY5372 and CY5381). At first, the index patient of each family underwent Next Generation Sequencing (NGS) that included the parallel analysis of a 5-gene panel, *COL4A3*, *COL4A4*, *COL4A5*, *CFHR5*, *FN1* (Ion Torrent, PGM, Life Sciences) [11]. Subsequently, WES was performed in the index patients plus one unrelated healthy subject by Macrogen (Kyoto, Japan), using the Illumina HiSeq platform (San Diego, CA, USA). The analysis included all exons, splice junctions and 5’-UTR/3’-UTR. Mean DNA fragment size was 101 bp. In total, 51.189.318 bases of genomic DNA were selected. The filtering algorithm of the data (Fig. 1) gave special attention to glomerulus specific genes [26]. Selected variants were validated by Sanger DNA re-sequencing using the ABI BigDye Terminator v1.1 Cycle Sequencing Kit and the ABI PRISM 3130*xl* genetic analyzer.

The study was approved by the Cyprus National Bioethics Committee and all participants gave a signed consent.

## Results

### WES analysis

NGS analysis for the 5-gene panel was negative. In family CY5500 (Table 1) a variant in the *COL4A5* gene, p.Asp654Tyr, was not considered as pathogenic and was overlooked as it did not involve a conserved glycine residue and was thought inadequate to explain the phenotype. WES resulted to a mean of 9000 non-synonymous SNPs and indels in coding regions, per individual (Additional file 1 After applying the analytical filtering algorithm (Fig. 1), we excluded about ~ 8500 variants and ended with a list of 549 candidate variants. When checked for any relation with the glomerular function, for animal models, or functional studies available in the literature, seven variants were considered of high risk, confirmed by Sanger re-sequencing (Tables 2 and 3). Two variants were predicted by five of six software to be damaging/disease causing. Specifically, variant *COL4A5*-p.Asp654Tyr was not found in 54 samples of the Cypriot general population nor in 305 samples with FMH tested at our setting. Also this variant was never reported in the ExAC genomes database [27]. Variant *LAMA5*-p.Pro1243Leu was neither found in 81 healthy individuals of the Cypriot general population nor was it present in any of 159 TBMN patients’ DNA. Also, according to the ExAC genomes database, it was found in European (non-Finish) populations with a frequency of ~ 1/21,123.

**Table 1:** Clinical data of CY5500 family

Patient	Gender	Micr. Hematuria	Proteinuria	CRF	Kidney Cysts	ESRD	Renal biopsy result (age range performed)
UCY2069	Male	YES	YES	YES	YES	YES, dialysis	FSGS – thick GBM (50–55 yrs)
UCY2075	Male	YES	YES	YES	YES	YES, transplanted	FSGS - no EM (50–55 yrs)
UCY2074	Female	YES	YES	NO	Not known	NO	FSGS (20–25 yrs)
UCY4041	Female	YES	YES	NO	NO	NO	FSGS – thinning and thickening of GBM (25–30 yrs)

*CRF* Chronic Renal Failure, *ESRD* End Stage of Renal Disease, *EM* Electron Microscope, *FSGS* Focal Segmental Glomerulosclerosis, *GBM* Glomerular Basement Membrane

**Table 2:** Effect prediction of the studied variants and general population information

Gene	Exon	DNA change	Aminoacid change	SNPs3D	SIFT	POLYPHEN2	Grantham	Mutation Taster	AlignGVGD	General population
*LAMA5*	30	c.3728 C > T	p.P1243L	1.19	0.02 (Damaging)	0.635 (Probably Damaging)	98	Disease Causing	C65 (High)	rs756101090 4/84492 (ExAC database)
*LAMB2*	27	c.4082 C > G	p.S1361 W	0.07	0.00 (Damaging)	0.999 (Probably Damaging)	177	Disease Causing	C65 (High)	–
*MMP24*	2	c.355 G > A	p.G119R	−1.84	0.07 (Tolerated)	0.999 (Probably Damaging)	125	Disease Causing	C65 (High)	3/60382 (ExAC database)
*SYNPO2L*	3	c.473 G > A	p.R158H	−0.70	0.01 (Damaging)	0.999 (Probably Damaging)	29	Disease Causing	C25 (Medium)	rs200006608 12/9758 (ExAC database)
*NID1*	14	c.2809 G > A	p.V937 M	1.61	0.04 (Damaging)	0.999 (Probably Damaging)	21	Polymorphism	C15 (Low)	rs200467845 11/59184 (ExAC database)
*TJP1*	5	c.428 G > A	p.R143Q	1.22	0.08 (Tolerated)	0.999 (Probably Damaging)	43	Polymorphism	C35 (Medium)	rs377122303 7/60349 (ExAC database)
*COL4A5*	26	c.1960 G > T	p.D654Y	−0.56	0.11 (Tolerated)	0.999 (Probably Damaging)	160	Disease Causing	C65 (High)	–

**Table 3:** Direct DNA re-sequencing results (AD: autosomal dominant). Variants not following AD inheritance have not been studied further

Family	Gene	Variant	WES result confirmed?	Ref. codon	New codon	Family analysis	Inheritance pattern compatible with AD	General population analysis
CY5500	*LAMA5*	p.P1243L	YES	CCG	CTG	YES	YES	0/81 0/159^a^
	*COL4A5*	p.D654Y	YES	GAT	TAT	YES	YES	0/54 0/305^a^
CY5372	*LAMB2*	p.S1361 W	YES	TCG	TGG	YES	NO	–
CY5381	*MMP24*	p.G119R	YES	GGG	GGA	YES	More samples needed	0/54
CY5394	*SYNPO2L*	p.R158H	YES	CGC	CTG	YES	More samples needed	–
	*NID1*	p.V937 M	YES	GTG	GTA	YES	NO	–
CY5417	*TJP1*	p.R143Q	YES	CGG	CAG	YES	NO	–

^a^Results from patients’ cohorts tested in our lab

### Family CY5500

**Fig. 2:**
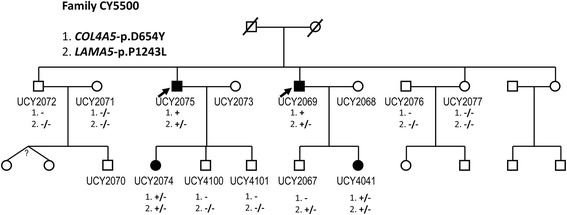
Pedigree of family CY5500. For family members with a UCY code, there is available a DNA sample. Four patients of the family carry both variants (patients UCY2075 and UCY2069 are actually hemizygous for the *COL4A5* variant). Family member UCY2067 carries only the *LAMA5* variant in heterozygosity, having normal kidney function and being negative for microscopic hematuria, aged 27

Both variants on *LAMA5* and *COL4A5* genes are co-inherited by all four patients in family CY5500. The *LAMA5* variant has been inherited by male UCY2067 that he is presently healthy. There is oral information that the grandfather (generation I) was suffering by chronic kidney disease. Below there is analytical clinical description for all mutation carriers of family CY5500 (Fig. 2).

#### UCY2069

**Fig. 3:**
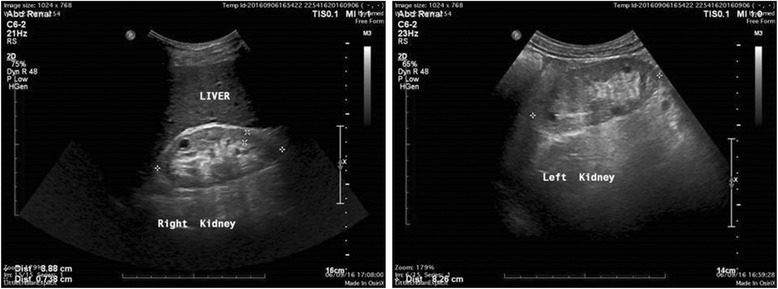
Ultra-sound of the kidneys of patient CA (UCY2069 on Fig. 1), aged 57-yrs, presently on ESRD. Both Kidneys are smaller than normal, measuring Rt kidney 8.88 cm in long diameter and the Lt kidney 9.51 cm, with increased echogenicity and cortical thinning, cortex measuring about 0.88 cm. There is no polycystic kidney disease (pelvocalyceal dilatation). Multiple cortical cysts are noted in both kidneys, about 6–8 cysts in each kidney, the bigger to the Rt about 4.5 cm and to the Lt about 3.6 cm. The cysts however have been present at least since 4-yrs, according to the patient’s medical record, at the time his renal function was much better (MDRD 50 ml/min)

CA is a 57-yr-old male, diagnosed with, and treated for, hypertension at the age of 36-yrs, associated with MH, minimal proteinuria and impaired renal function (MDRD eGFR = 66.0 ml/min/1.73m^2^). At the age of 54-yrs he developed heavy proteinuria of nephrotic range. Despite treatment with steroids and cyclosporine, his kidney function deteriorated and he developed ESRD at the age of 57-yrs. At the age of 54-yrs he had a kidney biopsy showing FSGS. The GBM was folded and appeared thick in many areas, while the podocytes appeared vacuolated and with marked fusion. Renal ultrasound showed multiple renal cortical cysts bilaterally, well before he reached ESRD (Fig. 3).

#### UCY4041

SCA, the daughter of patient CA, is a 25-yr-old girl with MH, borderline proteinuria, normal blood pressure and normal renal function (MDRD eGFR = 130 ml/min/1.73m^2^). A recent kidney biopsy showed FSGS. Mean width of the GBM was 218.73 nm, thinner than normal. Two measurements showed thickening alternating with thinning. In the most part the podocyte processes were maintained normal but there were a few segments with effacement. Folding of the GBM was observed in a percentage of 5% and in two areas thickening was observed. There were no clear pathognomonic features for Alport syndrome. Electron microscopy showed some deposits which most probably were IgM precipitates, recognized by immunofluorescent microscopy, localized in the periphery and the mesangium. Renal ultrasound was negative for renal cysts.

#### UCY2075

Male patient SA, brother of CA, aged 60-yrs, was diagnosed with heavy proteinuria, in the nephrotic range, associated with MH and renal impairment (MDRD eGFR = 43.0 ml/min/1.73m^2^) at the age of 51-yrs. At age 57-yrs he developed ESRD and had a cadaveric kidney transplantation. At the age of 51-yrs he had a kidney biopsy that revealed secondary FSGS, which at the time was attributed to pre-existing arterial hypertension and renal function impairment due to proteinuria. Electron microscope analysis is not available. Renal ultrasound showed multiple renal cortical cysts.

#### UCY2074

CSA, the daughter of patient SA, is a 34-yr-old girl, with MH and proteinuria. Her kidney function was normal at the age of 29-yrs, taking corticosteroids therapy (MDRD eGFR = 118.0 ml/min/1.73m^2^). She had a kidney biopsy at the age of 24-yrs, due to MH and proteinuria, revealing FSGS. Renal ultrasound was not available.

#### UCY2067

EA, the son of CA, aged 27, has inherited only the variant *LAMA5*-p.Pro1243Leu, and he is presently healthy.

## Discussion

Miner et al. [28] described *Lama5* (laminin alpha 5 chain) in 1995, member of the vertebrate subfamily of laminin chains, widely expressed in adult tissues, with highest levels in lung, heart, and kidney. In mature glomeruli, collagen α1α2α1(IV) and laminin α1β1γ1 (LN-111) interacting networks are replaced by collagen α3α4α5(IV), laminin α5β1γ1 (LN-511), and laminin α5β2γ1 (LN-521), as glomerular capillary loops expand [29]. It is documented that the maintenance of glomerular filtration barrier integrity requires laminin alpha5 [30]. Despite the fact that Lama5 mouse knockouts are fatal [31], mice with a hypomorphic *Lama5* mutation (Lama5neo) that reduces laminin α5 expression, exhibit proteinuria, hematuria and cystic kidneys [32]. Podocyte-specific inactivation of *Lama5* in mice, resulted in varying degrees of proteinuria and rates of progression to nephrotic syndrome. The GBM of the proteinuric mice appeared thickened and “moth-eaten,” and podocyte foot processes were effaced [30]. These facts, added to the population data mentioned above, enhance the likelihood that DNA variant *LAMA5*-p.Pro1243Leu is a mutation, probably a hypomorphic one, since the patients we identified exhibit a great phenotypic similarity with these animal models, presenting with hematuria, proteinuria, renal impairment, thinning and thickening of the GBM, podocyte foot processes effacement and multiple renal cysts.

In the absence of proper functional experiments it is impossible to know how exactly this Lama5 variant affects GBM structure and integrity. We can speculate however that the interaction of the laminin network with the collagen IV α3α4α5 network might be disturbed, resulting in distortion of the mature GBM meshwork and the glomerular filtration barrier. It is also reasonable to suspect an additive effect regarding the damage of GBM in the presence of a defective alpha 5 (Col-IV) chain, where if a dosage threshold is exceeded then a pathogenic phenotype appears. Even though analytical functional experiments are beyond the scope of this paper, we propose that the phenotype is better described by taking into consideration the variants at both loci than each one on its own [25].

It is equally worth mentioning that this is the third report presenting data supporting a probable contribution of the *LAMA5* gene to human disease. In a previous report the authors found novel *LAMA5* mutations, predicted to be deleterious, in three out of five patients with FSGS or presumed FSGS, also co-inherited with mutations in other genes [33]. Similar results were reported by another study [13]. No renal cysts were described in those patients. An additional argument in support of our hypothesis is the fact that laminin alpha 5 is an important protein of the glomerulus and an interacting partner of collagen IV, while animal models and patients exhibit similar phenotypes with our patients. While the evidence presented is valid, more studies are needed and more patients must be studied before this link is unequivocally documented. Probably, the easiest way is screening for mutations or for modifier variants in the *LAMA5* gene in multiple FMH and Alport syndrome families. Confirmation of these results will be of high clinical importance, expanding the spectrum of genes involved in glomerular pathologies. Finally, in recent years other examples have been reported of patients with *COL4A5* mutations and Alport syndrome, and phenotypes exacerbated by co-inheritance of mutations in other genes. These include mutations in other collagen IV genes (*COL4A3/A4*) and in the *MYO1E* gene [34, 35]. It should not escape our attention that high throughput analysis, including NGS technologies are enabling us to detect and connect clearly pathogenic or hypomorphic mutations in different genes and thus elucidating previously complex phenotypes. Often times though, it is difficult to discriminate cases of true non-Mendelian digenic inheritance from monogenic Mendelian inheritance of phenotypes exacerbated by serendipitous co-inheriting of a second non-allelic mutation or from the effect of otherwise neutral genetic modifiers, or even from the serendipitous co-inheritance of two independent clinical entities.

## Conclusions

In conclusion, we found evidence that digenic inheritance in patients of one family presenting with FMH, renal failure/ESRD, FSGS and cystic kidneys, can more clearly explain the phenotypic spectrum than one gene alone. The suspected genes are *COL4A5* and *LAMA5*. Existing animal models and two previous reports for rare mutations in *LAMA5* gene support this observation. These results need further confirmation, as *LAMA5* could explain a percentage of patients with complex phenotypes.

## Additional file

Additional file 1: WES statistics. Number of genetic variants called after the WES analysis. (DOCX 15 kb)
