# Supplementary material for: COL4A5 and LAMA5 variants co-inherited in familial hematuria: digenic inheritance or genetic modifier effect?
Source: BMC Nephrol. 2018 May 16;19:114. doi: 10.1186/s12882-018-0906-5 (PMC5954460; doi:10.1186/s12882-018-0906-5)
Supplement: Supplementary file 1 — WES statistics. Number of genetic variants called after the WES analysis. (DOCX 15 kb) [file 12882_2018_906_MOESM1_ESM.docx]

Suppl. Table 1: Mean number of the different types of genetic variants called after the WES analysis

| All SNPs | ~ 61,500 |
| --- | --- |
| Coding SNPs | ~ 19,300 |
| Synonymous SNPs | ~ 10,200 |
| Non synonymous SNPs | ~ 8,700 |
| Indels | ~ 5,000 |
| Indels in coding regions | ~ 400 |
